# Computational Intelligence for Studying Sustainability Challenges: Tools and Methods for Dealing With Deep Uncertainty and Complexity

**DOI:** 10.3389/frobt.2020.00111

**Published:** 2020-09-17

**Authors:** Edmundo Molina-Perez, Oscar A. Esquivel-Flores, Hilda Zamora-Maldonado

**Affiliations:** ^1^Tecnologico de Monterrey, Escuela de Ciencias Sociales y Gobierno, Monterrey, Mexico; ^2^Institute for Research in Applied Mathematics and Systems, Universidad Nacional Autonoma de Mexico, Mexico City, Mexico; ^3^Institute of Economics Research, Universidad Nacional Autonoma de Mexico, Mexico City, Mexico

**Keywords:** decision support tools, sustainability, end-of-century climate targets, computational intelligence, climate change, deep uncertainty

## Abstract

The study of sustainability challenges requires the consideration of multiple coupled systems that are often complex and deeply uncertain. As a result, traditional analytical methods offer limited insights with respect to how to best address such challenges. By analyzing the case of global climate change mitigation, this paper shows that the combination of high-performance computing, mathematical modeling, and computational intelligence tools, such as optimization and clustering algorithms, leads to richer analytical insights. The paper concludes by proposing an analytical hierarchy of computational tools that can be applied to other sustainability challenges.

## Introduction

The resolution of contemporary sustainability challenges requires the consideration of coupled systems, long-term time frames, multiple objectives, and deep uncertainty (Liu et al., 2013, 2016; Hull et al., 2015). For instance, sustainable ecosystem management, water planning, and climate change adaptation and mitigation require the joint consideration of environmental and human systems. These spheres (i.e., systems) are inexorably connected as changes in the behavior and constitution of the natural environment often induce changes in human institutions and incentives. Conversely, the evolution of human preferences, technology, and institutions determines significantly the development trajectories of natural resource systems. Often, if these interactions are not monitored and regulated, one or both systems stop functioning in a sustainable manner (Ostrom, 2009, 2011; Hull et al., 2015). For example, in the context of accelerated global climate change, if anthropogenic emissions continue rising, the growing concentration of greenhouse gases (GHG) in the atmosphere will result in climate imbalances (e.g., changes in precipitation patterns, higher temperatures) that can induce irreversible changes in natural ecosystems (e.g., loss of biodiversity) and in the economy (e.g., higher inequality).

Policy analysis in the context of sustainability is challenging. First, human and environmental spheres are complex systems: path dependencies in both require the consideration of large time frames, and their non-linear interactions induce dynamic behavior that is difficult to anticipate and characterize. Second, deep uncertainty affects both spheres as experts and stakeholders often disagree on the causal representation of these systems, the value of key parameters for analysis, and the relevance of different metrics for describing sustainability (Lempert, 2003; Marchau et al., 2019).

The combination of both conditions, complexity and deep uncertainty, has complicated the role of traditional policy analysis methods when applied to sustainability challenges. On the one hand, the use of simplistic models for analysis can result in omissions relevant for determining long-term outcomes. On the other hand, if the scope of an analysis is too narrow, it is difficult to make the analysis relevant to a wide range of stakeholders (Lempert, 2003; Marchau et al., 2019). Thus, a key emerging question in sustainability sciences is how to design robust policy interventions that explicitly account for complexity and deep uncertainty and which can inform in practical detail public policy discussions of sustainability challenges that affect a wide range of actors.

Modern computational intelligence tools, such as machine learning, optimization, agent-based modeling, and data visualization, offer opportunities for circumventing these limitations (Lempert et al., 2006; Groves and Lempert, 2007; Bryant and Lempert, 2010; Kasprzyk et al., 2013; Isley et al., 2015; Kwakkel, 2017). Yet, their analytical power for sustainability sciences can be best harnessed when these are used in an integrated way. For example, complex simulation models, such as agent-based models (ABMs), can be used as scenario generators in exploratory simulation contexts. Moreover, general purpose and multi-objective optimization techniques can be combined with ABMs to estimate the optimal policy response across large sets of feasible parametrizations. The resulting database can be further analyzed with machine learning algorithms to classify outcomes in terms of the combination of parameter values that trigger different policies. Finally, interactive data visualization techniques can be used to create decision support tools for stakeholders and the public.

Over the last two decades, a growing body of research has applied this integrative approach for studying various sustainability challenges in water (Lempert and Groves, 2010; Groves et al., 2019b; Molina-Perez et al., 2019), energy (Popper et al., 2009), and natural resource planning (Groves et al., 2016; Fischbach et al., 2019). The findings of these studies show that there are no silver bullets for achieving sustainability across human and environmental spheres and that policies that can contribute to achieving sustainable outcomes frequently rely on combinations of different measures that need to be implemented sequentially. First, by addressing immediate vulnerabilities through robust policies. Second, by responding adaptively to medium and long-term changes in both spheres (Groves et al., 2019b; Molina-Perez et al., 2019). This body of research, defined as Decision Making under Deep Uncertainty (DMDU) (Marchau et al., 2019), has cemented the foundations for the general application of computational intelligence tools to sustainability sciences.

This paper applies DMDU methods—specifically Robust Decision Making (Lempert, 2003; Groves et al., 2019b)—to structure an analysis of global climate change mitigation and to demonstrate that the combination of multiple computational tools for analyzing this sort of sustainability challenges leads to richer analytical insights than those produced by traditional monodisciplinary studies. Particularly, our analysis shows that by integrating optimization, integrated assessment models, and machine learning algorithms, it is possible to quantitatively identify key drivers of vulnerability of climate change mitigation policies. It also shows that alternative policy proposals can work as complements across regions to cost-effectively decarbonize the global economy. The paper concludes by proposing an analytical hierarchy of computational tools that can be applied to other sustainability challenges.

## Computer Modeling for Climate Change Policy Analysis

### Virtual Laboratories and Policy Regimes

Simulation models are popular tools in the field of climate change because of (a) the long-term time horizons needed to be taken into consideration, (b) the heterogeneous economic and technological conditions of countries and industries, and (c) the non-linearities and path dependencies associated with climate policy. To highlight how the combined used of integrated assessment models (IAMs) and other computational intelligence tools can result into a more detailed understanding of sustainability challenges, in this study we use the Exploratory Dynamic Integrated Assessment Model (EDIAM) developed by Molina-Perez (2016).

The EDIAM model is primarily based on the theoretical framework developed by Acemoglu et al. (2012), which takes into account the interrelation between climate mitigation, innovation, and growth. Particularly, it describes the propagation of climate policy impacts in the economy through endogenous productivity changes that affect labor, energy, and technology markets. EDIAM expands over this framework by including the role of learning-by-doing, differentiating technology properties across sectors, modeling entrepreneurs' investment decisions in continuous form, considering the role of technological transferability across nations, and calibrating environmental equations using a full ensemble of Coupled Model Intercomparison Project Phase 5 (CMIP5) climate projections (Taylor et al., 2012; IPCC, 2013).

The motivation for using EDIAM as an instrument for experimentation in this paper is based on four of its characteristics. First, it emphasizes the role that technology policy plays in climate mitigation. Second, it describes how climate policy propagates through time, changing the incentives of economic agents (i.e., path dependency). Third, it considers the interconnection between regions and between the environment and the economy and its role in shaping global outcomes (i.e., emerging behavior). Fourth, its specification allows for the exploration of a wide range of climate, economic, and policy assumptions. In short, this model serves as a good tool for analyzing how the interplay of complexity (i.e., emergent non-linear behavior) and deep uncertainty can be analyzed through the combination of different computational intelligence tools. Having said this, the reader should be conscious of the modeling features that fall outside the scope of this study. First, although empirically valuable, the model currently does not consider the possibility of endogenous innovation in the emerging region. Second, international trade and oil prices are also currently outside the scope of this work. Finally, in the optimization setup of this framework, without taxation on fossil fuels, it is not possible to mobilize the resources necessary to fund complementary technology policy for mitigating climate change. In reality, there are many other financial channels through which it would be possible to fund technology-based climate mitigation policies. In the following paragraphs, we describe the most relevant aspects of the model for this analysis^1^.

In EDIAM's framework, international climate policy is comprised on nine different elements:

1. Number of years policy intervention is active, starting in 2022: D
2. Carbon tax in the advanced region: τ^*A*^
3. Carbon tax in the emerging region: τ^*E*^
4. Technology subsidy for sustainable energy technologies in the advanced region: *h*^*A*^
5. Technology subsidy for sustainable energy technologies in the emerging region: *h*^*E*^
6. R&D subsidy for sustainable energy technologies in the advanced region: *q*^*A*^
7. R&D subsidy for sustainable energy technologies in the emerging region: *q*^*E*^
8. Green Climate Fund (GCF) technology subsidy for sustainable energy technologies in the emerging region: *h*^*G*^
9. GCF R&D subsidy for sustainable energy technologies in the emerging region: *q*^*G*^.

Formally, the optimal policy intervention is that which maximizes the intertemporal utility of representative consumers in the advanced and emerging regions (Equation 1.1)^2^. which depends both on consumption *C* (Equation 1.7) and the effects of fossil fuels used in production on temperature rise Δ*T* (i.e., quality of the environment *S*, Equations 1.3–1.6), subject to the intertemporal equilibrium conditions of both economies (Equations 1.8–1.13) and to the budget constraint (Equations 1.17, 1.18) in both regions (Acemoglu et al., 2012). The setup of the budget constraints is such that investments on technology-oriented climate action (i.e., technology and R&D subsidies) cannot be greater than the fiscal resources collected through a carbon tax in each region. Cooperation between regions is possible through the use of the GCF, which redirects resources from the advanced region to the emerging region.

Formally, this is expressed as follows^3^:

(1.1) $$\begin{array}{l} Max_{D,\;\tau^{A},\tau^{E},h^{A},h^{E},\;q^{A},q^{E},h^{G},q^{G}}\overset{T}{\underset{T_{0}}{\sum }}\frac{1}{{\left(1+\rho \right)}^{t}}\;\left(u^{A}+u^{E}\right) \end{array}$$

(1.2) $$\begin{array}{l} s.t.\\ u^{k}\left(C^{k},S\right)=\frac{{\left(\phi \left(S\right)C^{k}\right)}^{1-\sigma }}{1-\sigma }\; \end{array}$$

(1.3) $$\begin{array}{l} \phi \left(S\right)=\phi \left(\Delta S\right)=\frac{\begin{array}{c} {\left(\Delta T_{disaster}-\Delta T\left(S\right)\right)}^{\lambda }-\lambda \Delta T_{disaster}^{\lambda -1}*\\ \;\left(\Delta T_{disaster}-\Delta T\left(S\right)\right) \end{array}}{\left(1-\lambda \right)\Delta T_{disaster}^{\lambda }}\; \end{array}$$

(1.4) $$\begin{array}{l} \frac{dS}{dt}=-\xi \left({Y_{f}}^{A}+{Y_{f}}^{E}\right)+\delta \end{array}$$

(1.5) $$\begin{array}{l} CO_{2}=CO_{2|6.0{\;}^{{}^{\circ}}C}-S\; \end{array}$$

(1.6) $$\begin{array}{l} \Delta T=\beta *ln\left(\frac{CO_{2}}{CO_{2,0}}\right)\; \end{array}$$

(1.7) $$\begin{array}{l} C^{k}=Y^{k}-\psi_{s}\int_{0}^{1}x_{s,i}^{k}di-\psi_{f}\int_{0}^{1}x_{f,i}^{k}di\; \end{array}$$

(1.8) $$\begin{array}{l} Y^{k}={\left({Y_{s}^{k}}^{\frac{\varepsilon -1}{\varepsilon }}+{Y_{f}^{k}}^{\frac{\varepsilon -1}{\varepsilon }}\right)}^{\frac{\varepsilon }{\varepsilon -\;1}} \end{array}$$

(1.9) $$\begin{array}{l} Y_{j}^{k}={L_{j}^{k}}^{1-\alpha^{k}}\int_{0}^{1}{A_{ji}^{k}}^{1-\alpha^{k}}{x_{ji}^{k}}^{\alpha^{k}}di\; \end{array}$$

(1.10) $$\begin{array}{l} \frac{Y_{s}^{k}}{Y_{f}^{k}}={\left(\frac{p_{f}^{k}*\left(1+\tau^{k}\right)}{p_{s}^{k}}\;\right)}^{\varepsilon } \end{array}$$

(1.11) $$\begin{array}{l} \frac{p_{s}^{k}}{p_{f}^{k}}={\left(\frac{A_{f}^{k}}{A_{s}^{k}}\right)}^{\left(1-\alpha^{k}\right)}{\left(\frac{{\left(1-h^{k}\right)\psi }_{s}}{\psi_{f}}\right)}^{\alpha^{k}} \end{array}$$

(1.12) $$\begin{array}{l} \frac{\Pi_{s}^{k}}{\Pi_{f}^{k}}=\left(1+q^{k}\right)*\frac{\eta_{s}}{\eta_{f}}*\\ \frac{1}{{\left(1-h^{k}\right)}^{\frac{1}{1-\alpha^{k}}}}*{\left(\frac{\psi_{f}}{\psi_{s}}\right)}^{\frac{\alpha^{k}}{1-\alpha^{k}}}*{\left(\frac{p_{s}^{k}}{p_{f}^{k}}\right)}^{\frac{1}{1-\alpha^{k}}}*\frac{L_{s}^{k}}{L_{f}^{k}}*\frac{A_{s}^{k}}{A_{f}^{k}} \end{array}$$

(1.13) $$\begin{array}{l} \theta_{j}^{k}=\frac{e^{V(\Pi_{j}^{k})}}{\sum_{j=s}^{f}e^{V(\Pi_{j}^{k})}} \end{array}$$

(1.14) $$\begin{array}{l} \psi_{j}=\psi_{j,0}\;{\left(x_{j}^{A}+x_{j}^{E}\right)}^{\iota_{i}} \end{array}$$

(1.15) $$\begin{array}{l} \frac{dA_{j}^{A}}{dt}=\;\gamma_{j}\left(A_{j}^{A}\right)\eta_{j}\theta_{j}^{A}A_{j}^{A} \end{array}$$

(1.16) $$\begin{array}{l} \frac{dA_{j}^{E}}{dt}=\nu_{j}\gamma_{j}\left(A_{j}^{E}\right)\theta_{j}^{E}\left(A_{j}^{A}-A_{j}^{E}\right) \end{array}$$

(1.17) $$\begin{array}{l} \overset{D}{\underset{{t=D}_{0}}{\sum }}\left(h^{A}\psi_{s}\int_{0}^{1}x_{s,i}^{A}di\right)+\left(h^{G}\psi_{s}\int_{0}^{1}x_{s,i}^{E}di\right)+\left(q^{A}\eta_{s}\Pi_{s}^{A}\right)\\ +\left(q^{G}\eta_{s}\Pi_{s}^{E}\right)\leq \overset{D}{\underset{t=D_{0}}{\sum }}\tau^{A}p_{f}^{A}{Y_{f}}^{A} \end{array}$$

(1.18) $$\begin{array}{l} \overset{D}{\underset{{t=D}_{0}}{\sum }}\left(h^{E}\psi_{s}\int_{0}^{1}x_{s,i}^{E}di\right)+\left(q^{E}\eta_{s}\Pi_{s}^{E}\right)\leq \overset{D}{\underset{t=D_{0}}{\sum }}\tau^{E}p_{f}^{E}{Y_{f}}^{E} \end{array}$$

where

β: atmosphere's sensitivity to CO_2_ emissions (degrees Celsius)
ξ: atmosphere's carbon sink capacity (ppm/BTU/year)
δ: average rate of natural environmental regeneration (dimensionless/year)
Δ*T*: temperature rise since preindustrial times (degrees Celsius)
$CO_{2|6.0{\;}^{{}^{\circ}}C}$ : CO_2_ emissions concentration that will result in temperature rise of 6.0°C with respect to preindustrial levels^4^ (ppm)
Δ*T*_*disaster*_: 6.0 (degrees Celsius)
ϕ(*S*): costs of environmental quality degradation (dimensionless)
ε: elasticity of substitution (dimensionless)
ρ: discount rate (dimensionless/year)
$p_{j}^{k}$: primary energy prices of sector “j,” region “*k*” (usd/BTU)
$\Pi_{j}^{k}$: innovation profitability of sector “j,” region “*k*” (dimensionless)
α^*k*^: proportion of capital income to the total income of the economy in region “*k*” (dimensionless)
$\eta_{j}^{k}$: energy technologies propensity to innovation in sector “j,” in region “*k*” (dimensionless/year)
$\psi_{j,i}^{k}$: unitary cost of production for technology type “i” in sector “j” in region “*k*” (usd/machine)
$L_{j}^{k}$: share of labor working in sector “j,” region “*k*” (dimensionless)
$A_{j}^{k}$: productivity of sector “j,” region “*k*” (dimensionless)
$x_{ji}^{k}$: number of units of technology “i” used in sector “j” in region “*k*” (machines)
$\theta_{j}^{k}$: share of entrepreneurs working in sector “j,” region “k” (dimensionless)
γ_*j*_: mean R&D returns to productivity in sector “j” (dimensionless/year)
η_*j*_: innovation propensity of sector “j” (dimensionless/year)
ν_*j*_: probability of successfully imitating/adapting in the emerging region the technologies of sector “j” developed in the advanced region (dimensionless)
*T*: end of simulation, year 2100
*T*_0_: initial year of simulation, year 2012
*j* ∈ {“s”−*sustainble energy*− “f” −*fossil energy*−}
*k* ∈ {“A” −*advanced region*− “E” − *emerging region*−}.

As shown in Equation (1.2), we model consumer preferences through a constant relative risk aversion (CRRA) utility function, which depends both on consumption *C* and on the quality of the environment *S*. The parameter σ is the inverse of the intertemporal elasticity of substitution. Equation (1.3) describes the quality of the environment as dependent on temperature rise, which is determined by Equations (1.4)–(1.6), where Δ*T* represents the increase in average surface global temperature since preindustrial times for a given level of CO_2_ atmospheric concentration. The parameter λ controls how quickly the quality of the environment decreases as anthropogenic CO_2_ emissions rise. In the same fashion as Acemoglu et al. (2012), the state variable S is a metric of general environmental quality. In this study, this is empirically measured in parts per million (ppm) of atmospheric CO_2_ concentrations: the lower the value of S, the higher the environmental quality of the planet. The combination of Equations (1.3)–(1.6) connects this state variable to CO_2_ atmospheric concentrations, which in turn allows for internalizing the marginal impact of global fossil energy consumption on consumers' utility.

As shown in Equation (1.7), consumption depends on final production and the cost of technologies used in production. Final production (Equation 1.8) is modeled as a CES aggregate of the two primary energy sources: fossil fuel-based energy (f) and sustainable energy (s). Primary energy production (Equation 1.9) assumes that economic agents use labor and an infinite number of sector-specific technologies “i” for energy production (Acemoglu, 2002), $L_{j}^{k}$ represents the labor used in sector “j” ∈ {*f, s*}, $A_{ji}^{k}$ is the productivity of technology of type “i” used in sector “j”, and $x_{ji}^{k}$ is the number of units of technology type “i” in sector “j” used in production, in region “k.” For operationalizing the model, we rely on the same assumption used by Acemoglu et al. (2012): $A_{j}^{k}\equiv \int_{0}^{1}A_{ji}^{k}di$, such that $A_{j}^{k}$ is the average productivity of sector “j” in region “k.”

The share of production of each energy type “j” (Equation 1.10) depends on the prices of secondary energy types and the carbon tax. Secondary energy prices (Equation 1.11) in turn depend on productivity improvements in both energy sectors, technology costs, and technology subsidies. Technology costs (Equation 1.14) depend on the accumulated number of technologies used in each sector “j” in both regions. The parameter ι_*i*_ in this power-law function controls the rate at which experience leads to cost reductions in technology sector “i.” The evolution of productivity of section “j” in the advanced region (Equation 1.15) depends on share of entrepreneurs working in this sector, its R&D returns to productivity, and its innovation propensity. For the emerging region (Equation 1.16), we assume that technology entrepreneurs also innovate, but their efforts are targeted toward imitating the existing technologies in the advanced region. The success of these endeavors depends on the ease of transferability of technologies invented in the advanced region. The share of entrepreneurs working on sector “j” (Equation 1.13) determines the sectorial rate of technological progress, which depends on the value “*V*(.)” that investors assign to the mean profitability of sector “j” in region “k” ($\Pi_{j}^{k}$). Following the same approach as Train Kenneth (2003) and Achtnicht et al. (2012), this value function is a deterministic utility component that models economic agents' decisions over competing alternatives in the logistic form expressed in Equation (1.13).

The relative profitability of each sector “j” is described in Equation (1.12). If this ratio is >1, then the majority of research and development is directed toward sustainable energy technologies. In the tradition of Acemoglu (2002) and Acemoglu et al. (2012) framework, Equation (1.12) shows that there are three key forces determining which sector captures the greater share of entrepreneurial activity: (1) the “direct productivity effect” $\frac{A_{s}^{k}}{A_{f}^{k}}$ incentivizing research in the sector with the more advanced and productive technologies, (2) the “price effect” $\frac{p_{s}^{k}}{p_{f}^{k}}$ incentivizing research in the energy sector with the higher energy prices, and (3) the market size effect $\frac{L_{s}^{k}}{L_{f}^{k}}$ pushing R&D toward the sector with the highest market size. In addition to these forces, in the EDIAM modeling framework, two more factors are at play: (1) the “experience effect” ${\left(\frac{\psi_{f}}{\psi_{s}}\right)}^{\frac{\alpha }{1-\alpha }}$ pushing innovative activity toward the sector that more rapidly reduces technological production costs and (2) the “innovation propensity effect” $\frac{\eta_{s}}{\eta_{f}}$ incentivizing R&D in the sector that more rapidly yields new technologies. Note also that the research and technology subsidies also incentivize R&D in sustainable energy technologies. Finally, Equations (1.16) and (1.17) indicate that each region's contribution to the optimal policy should not be greater than the funds collected through the carbon tax.

Table 1 lists the set of policy regimes considered in this study. For each policy, we indicate in which sectors (i.e., carbon tax, technology subsidies, and R&D subsidies) cooperative actions are implemented and in which sectors individual independent actions are carried out. Thus, we model different policy regimes as a mix of individual and cooperative actions across sectors. In total, Table 1 describes nine different policy regimes. The future without action (FWA) represents the benchmark policy case in which climate policy is not implemented (i.e., laissez-faire economy). The policy regime “*P1. I. Carbon Tax [Both]*” represents a non-cooperative case in which both regions implement independently climate policy. Policy case “*P2. I. Carbon Tax* + *I.Tech-R&D[Both]*” depicts a different non-cooperative policy regime. In this case, the optimal policy response includes independent levels of taxation, technology subsidies, and R&D subsidies for both regions.

**Table 1:** Description of alternative policy regimes considered.

**Policy regime**	**Independent sectors**	**Cooperation sectors**	**Formalism in optimization problem**
P0 FWA: Future Without Action	• None	• None	τ^*A*^, τ^*E*^, *h*^*A*^, *h*^*E*^, *q*^*A*^, *q*^*E*^, *h*^*G*^, *q*^*G*^ = 0
P1 I. Carbon Tax [Both]	• Carbon tax	• None	τ^*A*^, τ^*E*^>0 *h*^*A*^, *h*^*E*^, *q*^*A*^, *q*^*E*^, *h*^*G*^, *q*^*G*^ = 0
P2 I. Carbon Tax + I.Tech-R&D[Both]	• Carbon tax •Technology subsidies •R&D subsidies	• None	τ^*A*^, τ^*E*^, *h*^*A*^, *h*^*E*^, *q*^*A*^, *q*^*E*^ > 0 *h*^*G*^, *q*^*G*^ = 0
P3 H. Carbon Tax + Co-Tech[GCF]+R&D[AR]	• No R&D subsidies in emerging region	• Harmonized carbon tax • Co-funded technology subsidies	τ^*A*^ = τ^*E*^ > 0 *h*^*A*^, *q*^*A*^ > 0 *h*^*E*^ = *h*^*G*^ > 0 *q*^*E*^ = *q*^*G*^ = 0
P4 H. Carbon Tax + Co-Tech[GCF] + I. R&D[Both]	• Independent R&D subsidies	• Harmonized carbon tax • Co-funded technology subsidies	τ^*A*^ = τ^*E*^ > 0 *h*^*A*^, *q*^*A*^, *q*^*E*^ > 0 *h*^*E*^ = *h*^*G*^ > 0 *q*^*G*^ = 0
P5 H. Carbon Tax + Co-R&D[GCF]+Tech[AR]	• No technology subsidies in emerging region	• Harmonized carbon tax • Co-funded R&D subsidies	τ^*A*^ = τ^*E*^ > 0 *h*^*A*^, *q*^*A*^ > 0 *q*^*E*^ = *q*^*G*^ > 0 *h*^*E*^ = *h*^*G*^ = 0
P6 H. Carbon Tax + Co-R&D[GCF]+I. Tech[Both]	• Independent technology subsidies in emerging region	• Harmonized carbon tax • Co-funded R&D subsidies	τ^*A*^ = τ^*E*^ > 0 *h*^*A*^, *h*^*E*^, *q*^*A*^ > 0 *q*^*E*^ = *q*^*G*^ > 0 *h*^*G*^ = 0
P7 H. Carbon Tax + Co-Tech-R&D[GCF]	• None	• Harmonized carbon tax • Co-funded R&D subsidies • Co-funded Technology subsidies	τ^*A*^ = τ^*E*^ > 0 *h*^*A*^, *q*^*A*^ > 0 *h*^*E*^ = *h*^*G*^ > 0 *q*^*E*^ = *q*^*G*^ > 0

*For each policy regime, it is indicated in which sectors (i.e., carbon tax, technology subsidies, and/or R&D subsidies) cooperative actions are implemented and in which sectors individual independent actions are carried out. Thus, each policy regime can be represented as a mix of individual and cooperative actions across sectors. The set of mathematical restrictions used to represent each policy regime in the optimization framework is noted*.

Multiple cooperation regimes are described in Table 1. For all these policy cases, we assume that regions agree initially on the implementation of a harmonized carbon tax as proposed by Nordhaus (2011); therefore, the carbon tax rate is the same across both regions. We also assume that cooperation under the GCF does not have to follow a unique architecture and that it is possible to cooperate in certain sectors, while allowing independent action in others. Policy case “*P3: H. Carbon Tax* + *Co-Tech[GCF]*+*R&D[AR]*” considers the case of a harmonized carbon tax across regions and cooperation in co-funded technology subsidies under GCF. However, in this case, independent R&D subsidies are only implemented in the advanced region. Policy “*P4: H. Carbon Tax* + *Co-Tech[GCF]* + *I. R&D[Both]*” expands on the latter case by considering that independent R&D subsidies are implemented in both regions.

Policy “*P5. H. Carbon Tax* + *Co-R&D[GCF]*+*Tech[AR]*” includes the implementation of a harmonized carbon tax in both regions, co-funded R&D subsidies under the GCF and independent technology subsidies in the advanced region. Policy regime “*P6. H. Carbon Tax* + *Co-R&D[GCF]*+*I.Tech[Both]*” expands policy case P5 by allowing for the implementation of independent technology subsides in both regions. Finally, policy regime “*P7: H. Carbon Tax* + *Co-Tech-R&D[GCF]*” considers the case in which in addition to a harmonized carbon tax, cooperation under the GCF includes co-funded R&D subsidies and technology subsidies.

### Uncertain Stressors Across Spheres

To analyze the performance of different policy regimes across uncertainty, we focus on four uncertain stressors, affecting two spheres: (1) the elasticity of substitution between fossil and sustainable energy inputs in production, and the economic agents' discount rate, impacting the economic sphere and (2) climate sensitivity to GHG emissions and the capacity of atmospheric carbon sinks affecting the ecological sphere. Thus, there are two types of elements in our analysis: (1) policy regimes that describe different sectorial interventions and cooperation schemes between regions and (2) scenarios which describe unique parameter combinations of economic and climatic variables.

**Figure 1:**
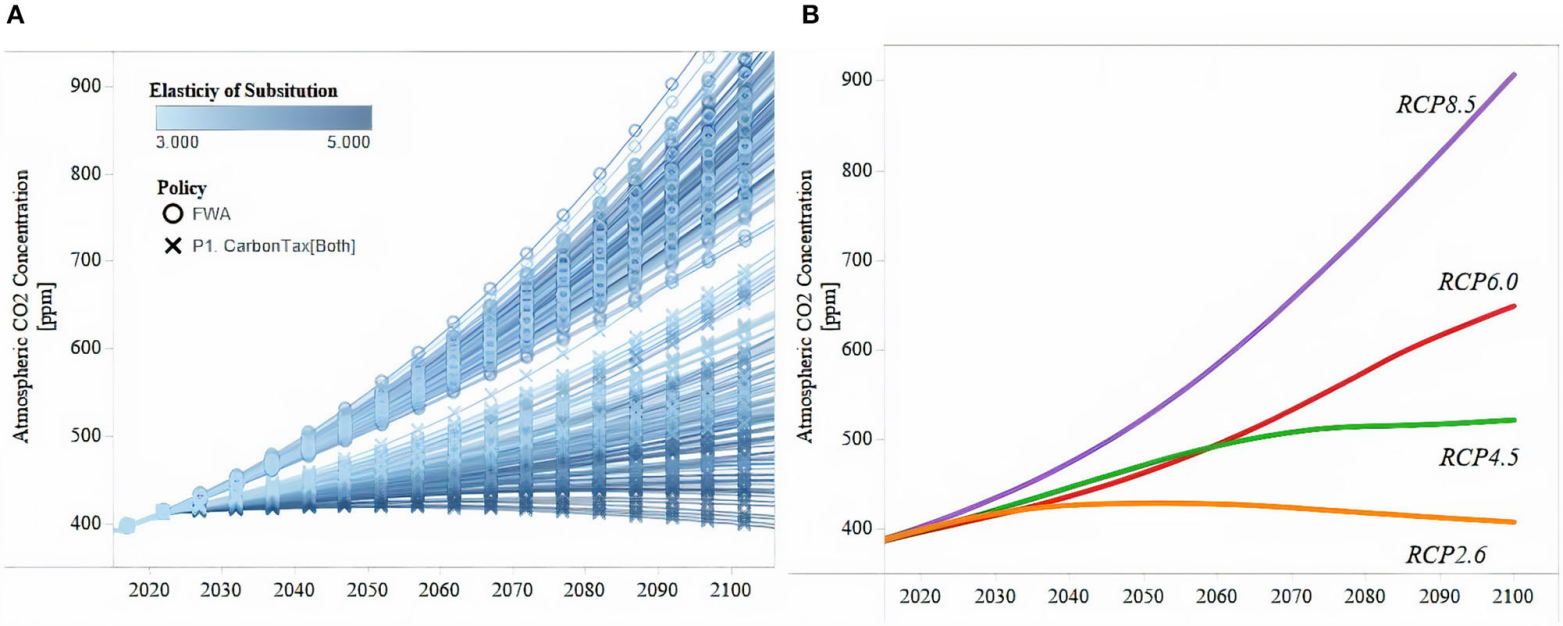
Comparison Between Simulated (Left Panel) and Original (Right Panel) RCP Emissions Trajectories. **(A)** Emissions Pathways (EDIAM simulations). **(B)** Emissions Pathways (RCPs in CMIP5). The right panel shows emissions time series for all RCPs considered in this study (i.e., RCP 2.6, RCP 4.5, RCP 6.0, and RCP 8.5). For each time step, the left panel shows the subset of emission trajectories used for comparing CMIP5 and EDIAM's output.

This framework allows us to explore uncertainty in more detail by generating an ample set of emission trajectories through variations of economic parameters and policy regimes. For example, Figure 1 compares a subset of simulated emission pathways that vary the elasticity of the substitution parameter (ε, Equation 1.8) for two policy regimes, against the four Representative Concentration Pathways (RCPs) included in the CMIP5 dataset. It is possible to see that the range of variation produced with these simulations is similar to that captured by the four RCPs included in CMIP5. This feature is important for this analysis because as discussed in section Machine Learning Algorithms for Identifying Decision-Relevant Conditions, by considering such a disaggregated set of variation, it is possible to identify with higher precision vulnerability thresholds.

The elasticity of substitution is an important parameter in the economic sphere because it describes the extent to which sustainable energy technologies can be used to substitute the functions of fossil energy technologies in secondary energy production. The results of Acemoglu et al. (2012) have spurred interest among empirical researchers on estimating more accurately the potential level of substitution between the two sectors. At present, initial empirical results show that the short- and long-term values of the elasticity of substitution are likely to be closer to the low substitution case considered in Acemoglu et al. (2012), but more importantly, these initial results show that the strength of the substitution effect in the long term is highly uncertain. For instance, Papageorgiou et al. (2013) use cross-country sectoral energy data and nested CES production functions to estimate this parameter. They find evidence that the elasticity of substitution in the short term is more likely to be in the low substitution range (ε = 3) of Acemoglu et al. (2012) study, but in the long term it is plausible that this parameter falls in the high-range values. Another study by Pottier et al. (2014) argues that the elasticity of substitution between sustainable energy and fossil energy is also likely to be in the low substitution range (ε = 3), perhaps even below one (ε < 1). They argue that this is the case mainly because capital stocks for most of the energy system last for many decades, and this delays substitution away from fossil energy. However, the authors consider that in the long term, as innovation broadens the range of technological possibilities, it is plausible that all energy sources will be fairly substitutable. These results and the debate among researchers on this topic support the notion that the elasticity of substitution between sustainable and fossil energy is a deeply uncertain parameter. These empirical findings show that the current state of science does not provide sufficient and adequate evidence to estimate accurately this parameter. They also suggest that in the long term a wide range of values is plausible.

**Figure 2:**
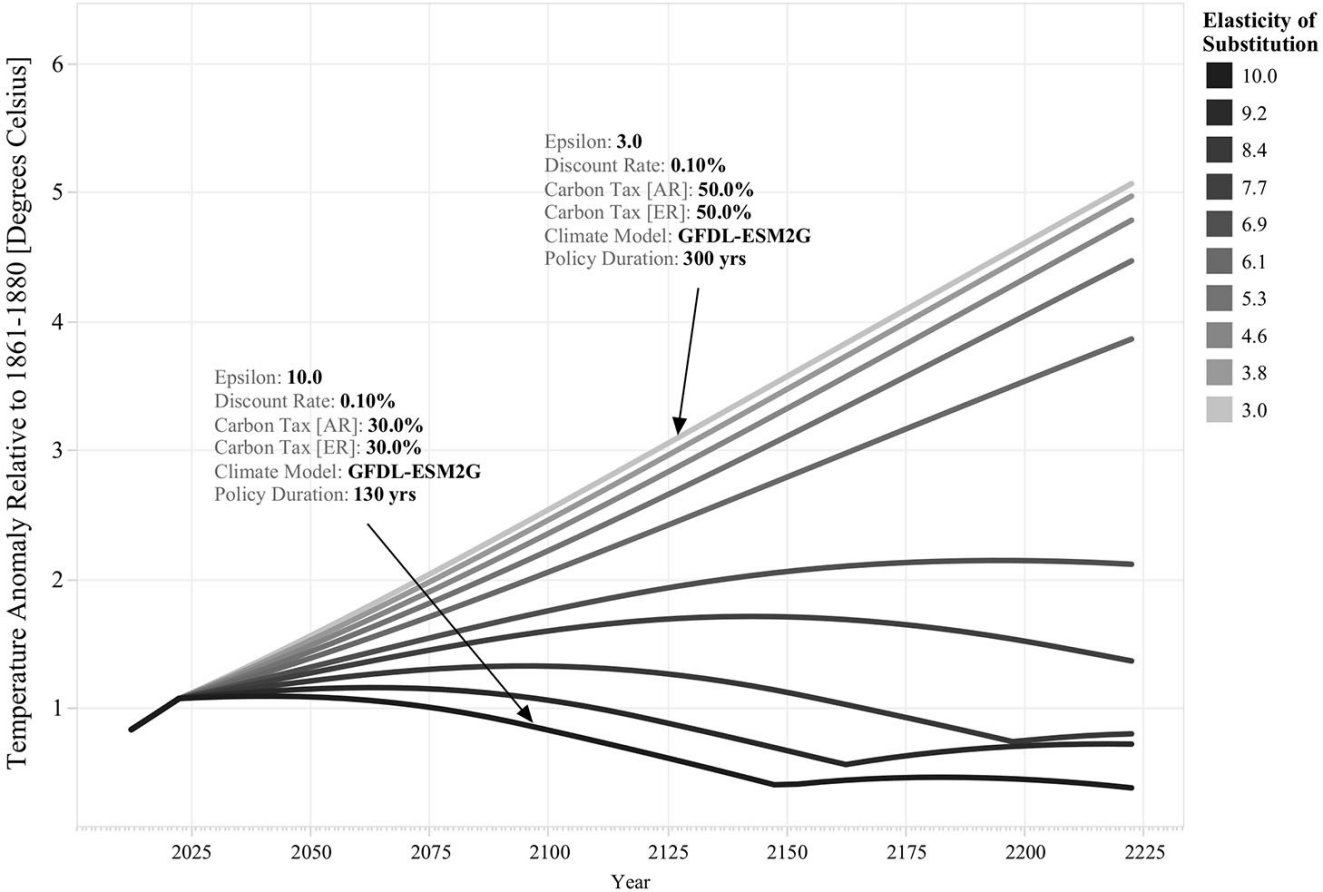
Effect of Different Discount Elasticities of Substitution Scenarios on Optimal Policy Response's Structure and Effectiveness. The vertical axis denotes temperature rise with respect to preindustrial levels for different simulated time series. The color legend indicates the level of substitutability between the two sectors: the darker colors denote scenarios of high elasticity of substitution, and the clearer-color scenarios of low elasticity of substitution. The structure of the optimal policy response, as well the climate scenario and discount rate parameters used for the simulations, is highlighted for the highest and lowest elasticity of substitution scenarios.

We explore the implications of varying levels of substitutability between the two sectors by considering 10 different scenarios for this parameter. Figure 2 lists the different elasticity of substitution scenarios considered in this analysis and exemplifies their effect on temperature rise stabilization. It shows that the 10 scenarios considered for the elasticity of substitution can result in substantially different outcomes. For instance, it shows that for three high levels of substitutability scenarios, ε = 10.0, ε = 9.2, and ε = 8.4, it is possible to induce a full self-reinforcing transition away from fossil energy before the end of the simulation runs (i.e., policy duration < 300 years). In contrast, for the low elasticity of substitution scenarios, ε = 3.0, ε = 3.8, ε = 4.6, and ε = 5.3, it is necessary to sustain policy intervention (i.e., harmonized carbon tax in both regions) during the entire simulation at a high level (i.e., 50%) to delay temperature rise. This shows that the cost and effectiveness of policy intervention is closely linked to the degree of substitutability between the fossil and sustainable energy sectors. The less substitutable these sectors are, the more effort is required to induce a successful transition toward sustainable energy and the decarbonization of secondary energy production in both regions.

The discount rate is a mathematical formalism that helps us express future costs and gains at today's equivalent value. In the context of climate change, this parameter attempts to describe how societies of today value the environmental and economic outcomes of the future. Controversy over the proper value of the discount rate lies at the heart of many of the debates associated with climate change policy. It should not be a surprise that studies that use different discounting values reach different conclusions regarding the structure of the optimal environmental policy required to stabilize global temperature rise. This debate is best exemplified by Nordhaus and Stern's research on the level of carbon taxation needed to keep temperature rise at sustainable levels (Acemoglu et al., 2012). In short, Nordhaus, using a discount rate of 1.50% per year, finds that an initial small carbon tax that increases over time would guarantee that temperature rise will be kept below three degrees Celsius in the long term, while Stern, using a discount rate of 0.10% per year, argues that a higher initial carbon tax is needed to achieve temperature rise stabilization sooner and avoid future significant damage from climate change. This disagreement among climate experts is evidence of the deep uncertainty associated with the discount rate.

In this analysis, we explore this uncertain stressor by considering a diverse set of discount rate scenarios. To develop these scenarios, we assume that the maximum value that this parameter can take is the one proposed by Nordhaus (i.e., 1.5% per year) and that the minimum value is the one proposed by Stern (i.e., 0.10% per year). However, we also consider three more possibilities in between to explore in more detail the role of varying levels of discounting on the structure of the optimal policy response.

**Figure 3:**
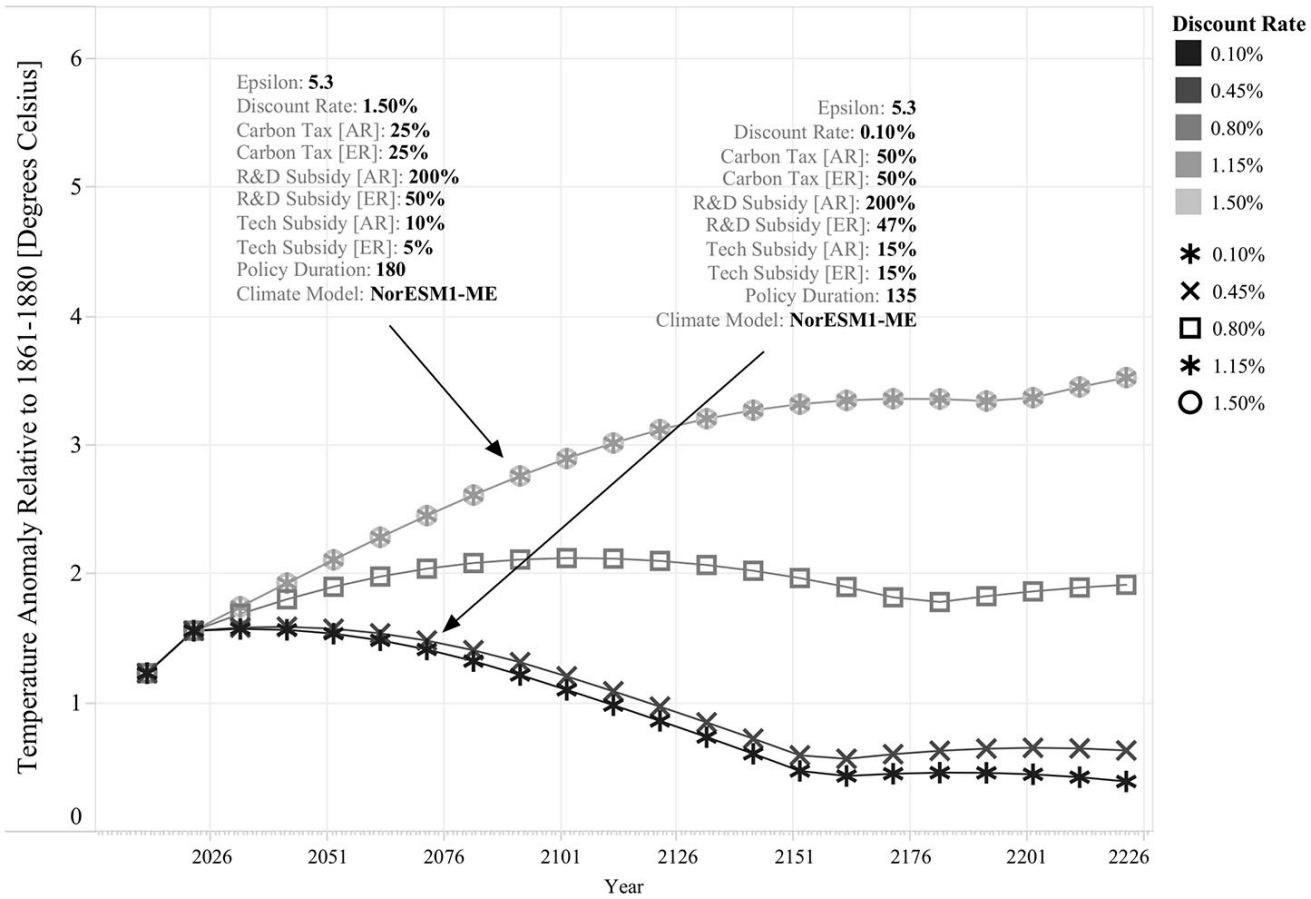
Effect of Different Discount Rate Scenarios on Optimal Policy Response's Structure and Effectiveness. Each line describes a single simulation run for a specific discount rate scenario. All other input parameters are held constant (ε = 5.3 and Climate Model = NorESM1-ME). The resulting optimal environmental policy is highlighted for two cases: for each, the different elements of the policy response are listed, including Carbon Tax, R&D Subsidies, Technology Subsidies, and Policy Duration for both regions. Note that the trajectory of the optimal policy response is the same for both the 1.15 and 1.5% discount rates.

Figure 3 lists the five discount rate scenarios considered in this analysis. This exercise provides an illustrative example of the discount rate's role in determining the structure of the policy response. By comparing the optimal policy response across the Stern (i.e., 0.10% per year) and Nordhaus (i.e., 1.5% per year) limits, it is possible to see that in the first case policy intervention is more decisive across both regions than policy intervention in the second case. For instance, the policy response with the 0.10% per year discount rate uses higher levels of carbon taxation and technology subsides in both regions. As a result, the environmental outcomes are also significantly different; for the 0.10% discount rate, temperature rise is kept below two degrees Celsius throughout the entire simulation, while for the 1.15% discount rate, temperature rise continues for over a century until it is stabilized at ~3°C. In this case, the cost of policy intervention is higher for the 0.10% discount rate, but it is important to note that in comparison to the 1.5% discount rate policy, this policy requires to be implemented during a shorter period of time (i.e., 135 vs. 180 years); thus, under alternative climate conditions, it is also feasible that both policies display similar intervention costs, or that in fact, the 0.10% discount rate policy becomes cheaper.

The uncertainty associated with the speed of temperature rise is associated with the limitations of our understanding of the global climate system. Each general circulation model used by the IPCC and included in the CMIP5 ensemble uses different assumptions and parameter values to describe the atmospheric changes resulting in growing anthropogenic GHG emissions, and, as a result, the magnitude of the estimated changes varies greatly among different modeling groups. In this respect, one of the features of EDIAM is that it uses 12 GCMs included in the CMIP5 data ensemble to calibrate the parameters ξ, δ, β, and S_0_ in Equations (1.4) and (1.6). Thus, in EDIAM, GCMs are described as unique combinations of climate sensitivity of GHG (β) and the capacity of the atmospheric carbon sink (δ, S_0_) as listed in Table 2.

**Table 2:** Estimated climate parameters using CMIP5 GCM models.

**Climate scenario**	**β**	**ξ**	**δ**	**S_**0**_**
MIROC-ESM-CHEM	6.13	0.010	0.00278	590
GFDL-CM3	6.11	0.010	0.00259	635
MIROC-ESM	5.93	0.010	0.00260	633
bcc-csm1-1	5.00	0.010	0.00182	916
MPI-ESM-LR	4.67	0.010	0.00161	1,042
MPI-ESM-MR	4.67	0.010	0.00161	1,045
NorESM1-ME	4.34	0.010	0.00136	1,236
MRI-ESM1	4.26	0.010	0.00130	1,294
NorESM1-M	4.13	0.010	0.00119	1,415
MIROC5	4.12	0.010	0.00119	1,417
GFDL-ESM2M	3.29	0.010	0.00071	2,403
GFDL-ESM2G	3.19	0.010	0.00063	2,695

*The table lists the estimated parameters for the 12 CMIP5 climate models included in this study; the parameters of Equations (1.4) and (1.6) are listed for each climate model. These parameters are estimated using CO_2_ emission levels' variation across representative concentration pathways (RCPs) for each of the climate models in an autoregressive model*.

**Figure 4:**
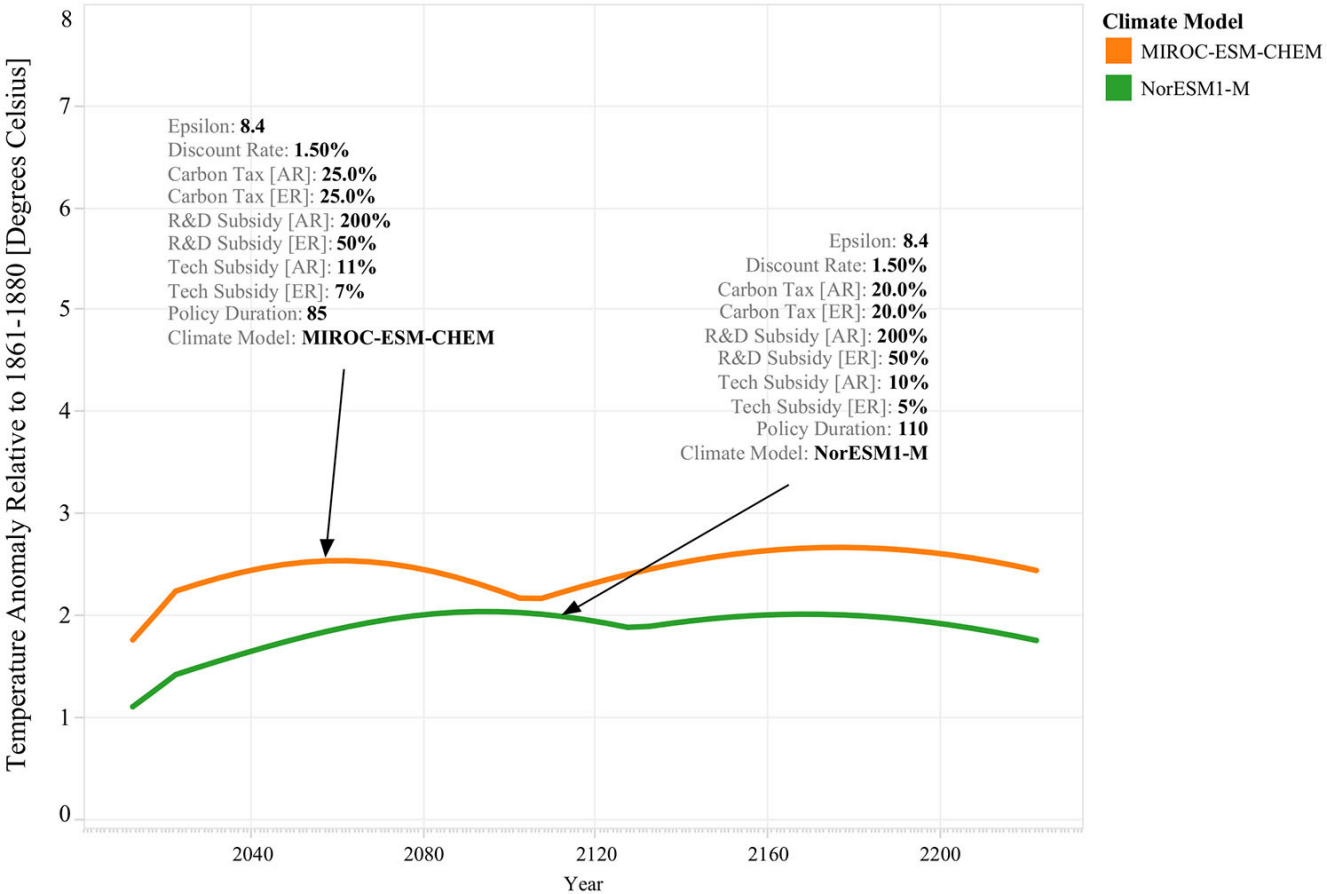
Effect of Different Climate Scenarios on Optimal Policy Response's Structure and Effectiveness. This figure shows temperature rise time series for two simulation experiments. Both simulations used the same parameter values for the elasticity of substitution (i.e., 8.4) and the discount rate (i.e., 1.50% per year) but are run for different climate scenarios: MIROC-ESM-CHEM (i.e., orange line) and NorESM1-M (i.e., green). For each simulation, the pointing arrows indicate the resulting optimal policy as a combination of carbon taxes, research subsidies, and technology subsidies across both regions.

Figure 4 provides an illustrative example of how different GCMs may lead to a different structure of the optimal environmental policy. It shows that for a GCM that displays higher climate sensitivity, such as *MIROC-ESM-CHEM*, it is possible that under certain circumstances, the optimal policy uses a higher mix of carbon taxes, research subsidies, and technology subsidies than in the case of a GCM that displays lower climate sensitivity, like *NorESM1-M*. It also shows that the environmental outcomes between both scenarios are different: in this case, for both simulation runs temperature rise is successfully mitigated, but this occurs at a higher level for climate scenario *MIROC-ESM-CHEM* than for scenario *NorESM1-M*. It also shows that the cost of policy intervention is unambiguously higher for climate scenario *MIROC-ESM-CHEM* because although the rate of carbon taxation is smaller in climate scenario *NorESM1-M*, policy intervention lasts longer in the latter case. Evidently, these results can change when combined with other uncertainties, yet it offers an illustrative example of the interplay between the optimal policy response and the different climate scenarios.

## Using Models Differently Through Computational Experimentation

### Considering Multiple Dimensions of Merit

Sustainability challenges often deal with multiple spheres (e.g., economic, ecological, technological) (Liu et al., 2013; Hull et al., 2015); as a result, sustainability studies need to deal with multiple, and often, opposing measures of merit. Climate change mitigation offers a clear example of this as it requires the consideration of different metrics to evaluate and compare the performance of competing policy proposals. In this study, we focus primarily on the outcome that policy intervention has on economic and environmental conditions by the end of the century. This aligns the scope of this work to discussions associated with the end-of-the century temperature rise and emission stabilization targets.

From an economic perspective, we estimate the cost of policy intervention by comparing consumption levels across the policy intervention case and the laissez-faire economy. Then, the higher the reduction in consumption compared to the laissez-faire economy, the higher the costs of policy intervention. From an environmental perspective, we consider two metrics: the end-of-the century temperature rise level and end-of-century CO_2_ atmospheric concentrations. The first metric is useful for comparing policies in terms of the temperature levels that are plausible with its implementation. The second metric is useful to analyze whether or not a policy stabilizes CO_2_ emissions such that temperature permanently stops rising. We make this distinction because maintaining temperature rise below a certain threshold (e.g., two degrees Celsius) does not entail that atmospheric CO_2_ concentrations are also stabilized. Without stabilization, if climate policy is lifted, temperatures will continue rising.

### Experimental Design and Case Generation

We use the elements outlined in the previous sections to conduct several simulation experiments. The experimental design includes a full-factorial sampling design across different EDIAM's parameters; this includes

- 12 climate scenarios
- 10 elasticity of substitution scenarios
- 5 discount rate scenarios.

We considered all possible combinations of these uncertain exogenous factors for developing individual model parametrizations, which yields a total of 600 cases. Table 3 summarizes the scope of the experimental design of this study using the XLRM framework developed by Lempert (2003), while emphasizing that we are dealing specifically with uncertain stressors in the context of sustainability (i.e., XSLRM).

**Table 3:** XSLRM summary of experimental design.

**Uncertain stressors (XS)**	**Policy levers (L)**
Climate uncertainty: • 12 Climate scenarios Economic uncertainty: • 10 elasticity of substitution scenarios • 5 discount rate scenarios	• P0. FWA (Future Without Action) • P1. I. Carbon Tax [Both] • P2. I. Carbon Tax + I.Tech-R&D[Both] • P3. H. Carbon Tax + Co-Tech[GCF]+R&D[AR] • P4. H. Carbon Tax + Co-Tech[GCF] + I. R&D[Both] • P5. H. Carbon Tax + Co-R&D[GCF]+Tech[AR] • P6. H. Carbon Tax + Co-R&D[GCF]+I. Tech[Both] • P7. H. Carbon Tax + Co-Tech-R&D[GCF]
**System relationships (R)**	**Metrics (M)**
• Exploratory dynamic integrated assessment model (EDIAM)	• End-of-century temperature rise • Stabilization of GHG emissions • Economic costs of policy intervention

*The main components of the exploratory analysis are grouped according to four different categories: (1) the deep uncertainty scenario taken into account (i.e., 12 climate scenarios, 10 Elasticity of Substitution Scenarios, and 5 Discount Rate Scenarios), (2) the policy regimes analyzed (i.e., 8 different policy regimes, (3) the system relationship that links actions to consequences (i.e., EDIAM model), and (4) the metrics considered to analyze the performance of different policies*.

## Machine Learning Algorithms for Identifying Decision-Relevant Conditions

### Experimental Datasets and Results

**Figure 5:**
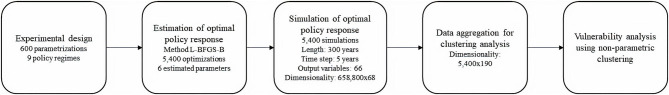
Sequence Used for Data Production and Analysis. Blocks denote analytical steps used for producing the datasets for this analysis. The general characteristics of the methods and datasets used in the analysis are indicated in each box.

Figure 5 describes the sequence of steps we implemented to produce the datasets used for the analysis described in this section. For each of the steps in the process, this figure indicates the method and general characteristics of the datasets produced. The experimental design consisted of 5,400 optimization runs across 600 parametrizations that vary climate parameters, elasticity of substitution, and the discount rate. The optimization runs estimate the optimal policy response for each of the parametrizations, considering the restrictions of the different policy regimes, using Byrd et al. (1995) “L-BFGS-B” method for constraint optimization. On average, it takes 10,000 simulation runs to converge on a solution for the optimization problem. Thus, in total, the results described in the following sections required ~54 million simulation runs.

Four datasets are relevant for this sequence of steps. The experimental design dataset describes how the combination of climate and economic parameters vary across the different optimization runs. In terms of its cardinality, there are 600 unique combinations of parameters in this dataset, identified by unique future ids, which are combined with the 9 policy regimes, indicated by a unique policy id. The optimal policy response dataset describes for each of the 5,400 runs the combination of policy parameters that solves the optimization problem described in section Virtual Laboratories and Policy Regimes; each of these optimal vectors is unique, since each of the estimated variables is continuous (e.g., carbon tax rates, subsidy rates, and R&D intensities). The simulation dataset describes the dynamic behavior of the system under these 5,400 optimal policy vectors using 66 output variables of the EDIAM model. Finally, in the scenario discovery dataset, we aggregate simulation results by summarizing the dynamic behavior of each run using an expanded set of variables that compare absolute and relative behavior across regions and sectors. For instance, by comparing end-time technological progress with respect to initial conditions, technological progress in competing sectors within regions, and technological progress across regions.

**Figure 6:**
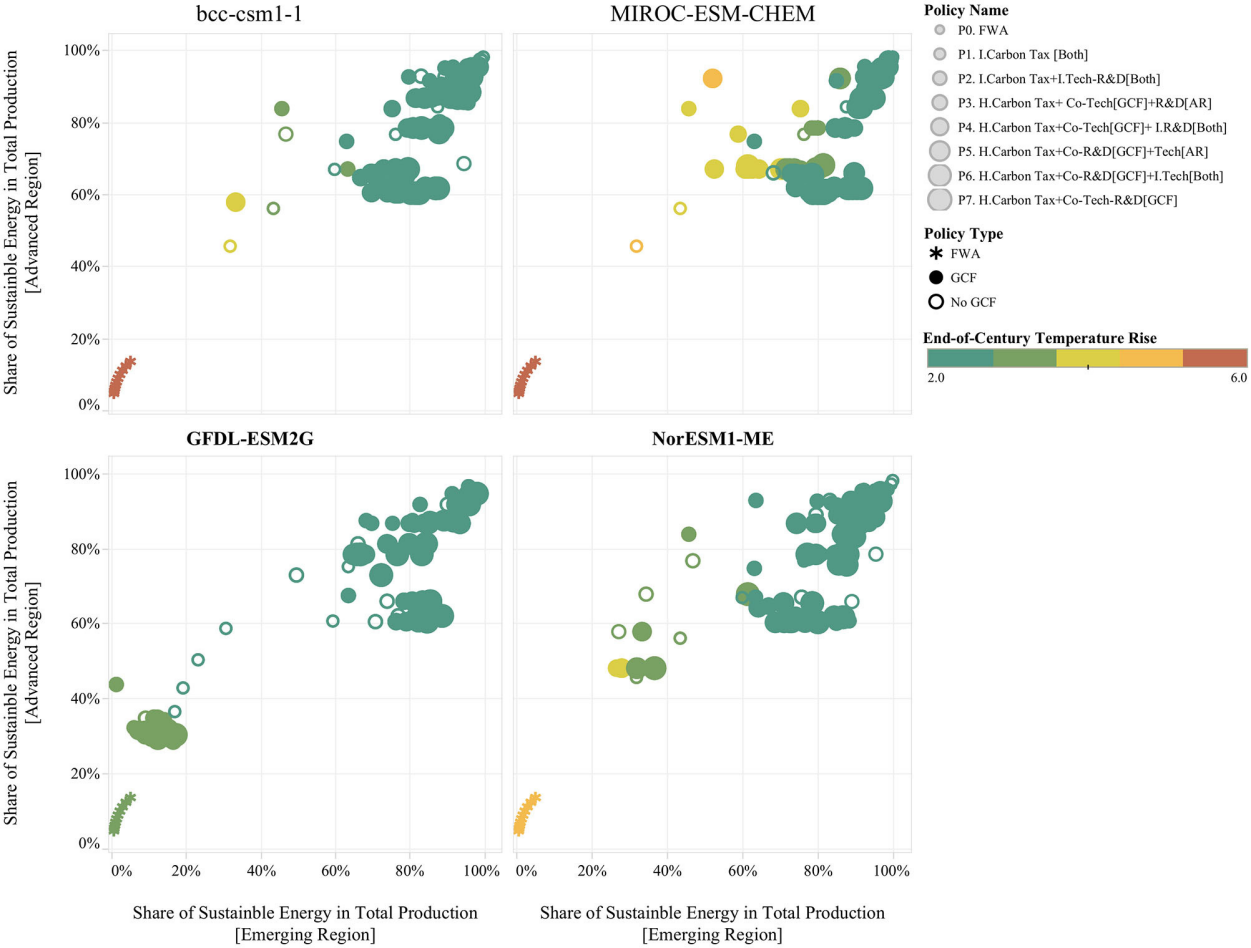
Example of Experiment Results across Different GCMs. Each point describes an individual future through the penetration of sustainable primary energy as percent of total secondary energy production for the emerging region (x-axis) and for the advanced region (y-axis). The size of the points reflects the type of policy regime, the smaller points denote policy regimes which include the FWA and the two non-GCF policies (i.e., P1 and P2), and bigger points indicate GCF-based policies (i.e., P3, P4, P5, P6, and P7). The color legend denotes end-of-the century temperature rise; the green points describe temperature rise conditions closer to the 2°C target, while the red points describe temperature rise conditions closer to the environmental disaster condition of 6°C. The figure includes four panes; each pane displays results for a different GCMs.

Results from the simulation runs generated by the experimental design are shown in Figure 6. These results are useful for highlighting some of the features of the system and of the policy response. The figure shows that there is ample variation with respect to the penetration levels of sustainable energy that can be achieved through the various policy regimes. It is possible to see that the FWA (i.e., laissez-faire economy) results in limited penetration of sustainable energy across both regions, and as a result, end-of-century temperature rise levels are close to the environmental limit (i.e., 6°C). Figure 6 also reveals that the best environmental outcomes are concentrated in the upper right corner of these panes. These futures represent scenarios in which the policy response induces a successful transition toward sustainable energy across both regions. It is possible to see that the non-GCF policies (i.e., P1 and P2) can achieve similar levels of penetration of sustainable energy in both regions than GCF-based policies (i.e., P3–P7). These results also show that policy performance varies across GCMs in terms of the penetration of sustainable energy and the resulting temperature rise.

**Figure 7:**
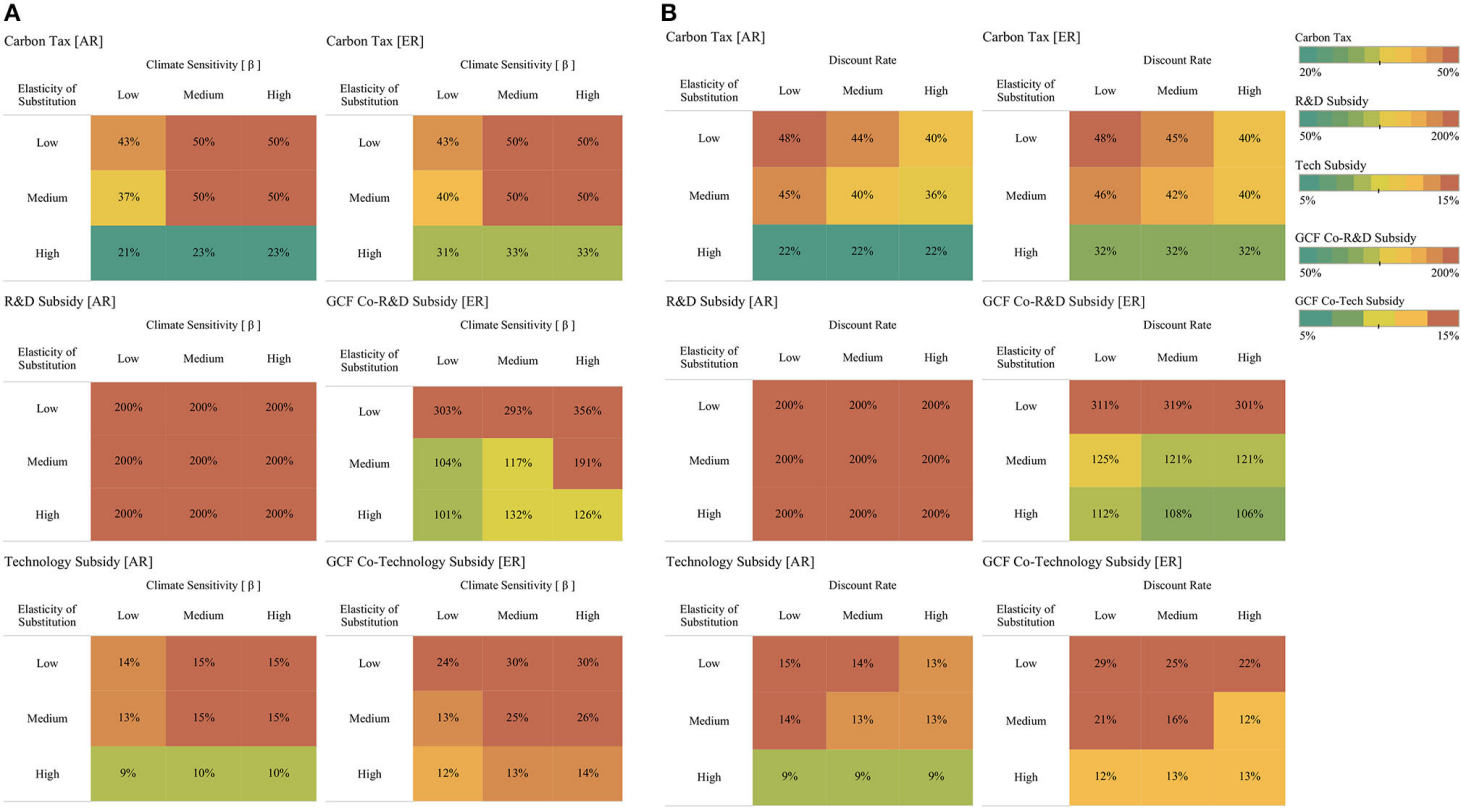
Changes in Optimal Response's Structure Across Different Elasticity of Substitution, Climate Sensitivity, and Discount Rate Scenarios. P2: I. Carbon Tax + I. Tech-R&D [Both]. For each panel **(A,B)**, the left panes describe changes in the structure of the optimal policy in the advanced region (AR) and the right panes describe changes in the emerging region (ER) for the independent comprehensive policy regime (P2). The top panes show results for the individual carbon taxes, the middle panes for R&D subsidies, and the bottom pane for technology subsidies. Uncertainty values are described using three bins; for the elasticity of substitution, these bins are defined as low, medium: [5:8), and high: [8:10); for climate sensitivity, these bins are defined, considering the range of values presented in Table 2, as low: [3:5), medium: [5:6), and high: [6,7); and for the discount rate, these bins are defined as low: [0, 6%), medium [6,12%), and high [12,15%). The legend of each cell represents the mean value of the policy element for the subset of futures describe by the intersecting bins (elasticity of substitution, climate sensitivity, and discount rate). The color legend denotes the effort level of the policy response: colors toward red denote higher effort policies; colors toward green denote lower effort policies.

The structure of the optimal environmental policy varies across the uncertainty space in order to meet the climate policy targets described. This variation in the structure of the optimal response has important implications for policy design and exemplifies the richness of the experimental results. For example, Figure 7A shows for policy P2: I. Carbon Tax + I. Tech-R&D [Both] how the optimal response changes across two parameters: the elasticity of substitution and climate sensitivity to GHG. The left panes describe changes in the structure of the optimal policy in the advanced region (AR), and the right panes describe changes in the emerging region (ER). The top panes show results for the individual carbon taxes, the middle panes for R&D subsidies, and the bottom pane for technology subsidies. The results presented in this figure show that the structure of the optimal policy is very sensitive to the combined effect of the elasticity of substitution and climate sensitivity: the higher the climate sensitivity and the lower the elasticity of substitution, then the higher the effort of the optimal policy response.

The discount rate is another important factor that influences the structure of the optimal policy response. Figure 7B describes changes in the structure of the optimal policy across different scenarios of the elasticity of substitution and the discount rate. As expected, it shows that the strength of the policy response increases as the discount rate diminishes. However, in this case it is possible to see that as the elasticity of substitution increases, the influence of the discount rate in the structure of the optimal policy diminishes. For high elasticity of substitution scenarios, it is possible to see that the structure of the optimal policy is insensitive to changes in the discount rate. These results highlight the importance of regional differences in defining the structure of optimal environmental regulation. It is possible to see that in the emerging region carbon taxation is always equal or higher than carbon taxation in the advanced region. In contrast, the technology policy elements of optimal environmental regulation are higher in the advanced region than in the emerging region. Since technologies are developed in the advanced region, then the optimal policy prioritizes accelerating technology development over taxation in this region, while in the emerging region, higher taxation creates a strong market niche for sustainable energy, which is used more effectively by R&D and technology subsides that accelerate the technological catching-up process.

A similar analysis for policy regime P7 “*H. Carbon Tax* + *Co-Tech-R&D[GCF]*” shows that under the GCF the level of carbon taxation reduces for both regions compared to the level of taxation in the non-cooperative policy regime (i.e., P2). Additionally, the optimal level of effort in R&D and technology subsidies in the emerging region is on average higher than the optimal level of effort in the non-cooperative policy regime. This indicates that under the GCF, it is feasible for the emerging region to make higher investments in R&D and technology subsidies and reduce the rate of taxation. Similarly, for the advanced region, these results show that it is possible to reduce the level of carbon taxation by co-funding R&D and technology subsidies in the emerging region. Finally, the results show that in the most adverse scenarios under the GCF (i.e., low elasticity of substitution and high climate sensitivity), optimal environmental regulation requires higher R&D and technology subsidies in the emerging region than in the advanced region.

### Machine Learning Algorithms for Describing Vulnerability Conditions

The previous section describes general characteristics of the experimental datasets and insights of the computational experiment. Yet, these results do not provide a systemic understanding of how the interaction of the set of stressors considered in the experiment affect the structure and effectiveness of optimal climate policy response under uncertainty. To address this, we follow two steps. First, we classify experimental outcomes with respect to whether or not specific policy objectives are met. Second, we use non-parametric clustering analysis for understanding the combination of factors that lead to meeting these objectives. We consider an outcome is not vulnerable when the temperature target (i.e., 2°C) and/or the stabilization targets are met. This suggests that there are two outcome types of interest in this experiment:

1. Simulations in which the 2°C end-of-century temperature rise target is met
2. Simulations in which the 2°C end-of-century temperature rise target and CO_2_ stabilization are met.

Table 4 presents the performance statistics of different policy regimes across the 600 parametrization cases considered for these two outcome types. As expected, the FWA does not meet any of the climate change objectives. It also shows that for the independent carbon tax policy (i.e., P1) in the majority of simulations, it is possible to keep the temperature rise below 2°C, but in none of these cases is this policy able to stabilize CO_2_ emissions. This shows that this policy is effective in delaying temperature rise but is less effective at inducing successful decarbonization across regions. In contrast, policies that complement carbon taxes with R&D and technology subsidies are able to meet the CO_2_ stabilization targets in a higher number of futures. It is possible to see that the stabilization targets are met in less than one third of the futures considered. In this respect, some of the GCF-based policies (i.e., P4 and P7) are slightly more effective than the non-GCF policy (i.e., P2) in meeting the stabilization target.

**Table 4:** Performance of optimal policy response across different policy regimes.

**Policy name**	**Number (percentage) of futures meeting the end-of-century climate policy target**	
	**Temperature rise below 2°C**	
	**CO_**2**_ stabilization achieved**	**CO_**2**_ stabilization not achieved**
P0. FWA	0 (0)	0 (0.0)
P1. I. Carbon Tax [Both]	0 (0)	375 (62.5)
P2. I. Carbon Tax + I. Tech-R&D[Both]	153 (25.5)	398 (66.3)
P3. H. Carbon Tax + Co-Tech[GCF] + R&D[AR]	130 (21.7)	344 (57.3)
P4. H. Carbon Tax + Co-Tech[GCF] + I. R&D[Both]	153 (25.5)	391 (65.2)
P5. H. Carbon Tax + Co-R&D[GCF] + Tech[AR]	130 (21.7)	395 (65.8)
P6. H. Carbon Tax + Co-R&D[GCF] + I. Tech[Both]	145 (24.2)	415 (69.2)
P7. H. Carbon Tax + Co-Tech-R&D[GCF]	165 (27.5)	402 (67.0)

*The table summarizes the performance of each policy across the 600 parametrizations considered for the four outcome types. The numbers (percentage) of parametrization meeting the different end-of-century climate policy targets are listed under each column*.

For the second step, we use the algorithm PRIM (Patient Rule Induction Method) (Friedman and Fisher, 1999), a non-parametric bump hunting classification algorithm, to quantitatively describe vulnerability condition of different policies. In particular, we use PRIM in the context of the scenario discovery method developed by Bryant and Lempert (2010). Thus, for each policy regime, we classify simulation outcomes into two cases of interest (*I*_*s*_): (1) cases in which the policy target is met and (2) cases in which the policy target is not met. Then, PRIM is used to parse the simulation database into concise clusters that describe dimensional conditions under which policies do not meet targets. This is done through the estimation of recursive peeling trajectories, as class types often require more than one cluster to be fully described. This implies that once an initial cluster is chosen, the algorithm removes all the data points from the dataset inside the first cluster and replicates the peeling/pasting process with the remaining data.

Two statistical measures are used to describe the suitability of a decision relevant cluster. Coverage (Equation 2) measures how completely the cases defined by cluster B cover the cases of interest (*I*_*s*_); in this study, this is the percent of total vulnerable cases that are captured by the cluster. Density (Equation 3) measures the purity of the scenarios; in this study, this is the percent of cases within the cluster that are vulnerable. Interpretability of these cluster is an important subjective measure; generally, the fewer dimensions used by the cluster, the higher its suitability for the analysis.

(2) $$\begin{array}{l} Coverage=\frac{\sum_{x_{i}\in B}y_{i}^{\prime }}{\sum_{x_{i}\in x^{I}}y_{i}^{{}^{\prime }}}) \end{array}$$

(3) $$\begin{array}{l} Density=\frac{\sum_{x_{i}\in B}y_{i}^{\prime }}{\sum_{x_{i}\in B}1\;} \end{array}$$

where $y_{i}^{\prime }=1$ if *x*_*i*_ ∈ *I*_*s*_ and $y_{i}^{\prime }=0$ otherwise.

**Figure 8:**
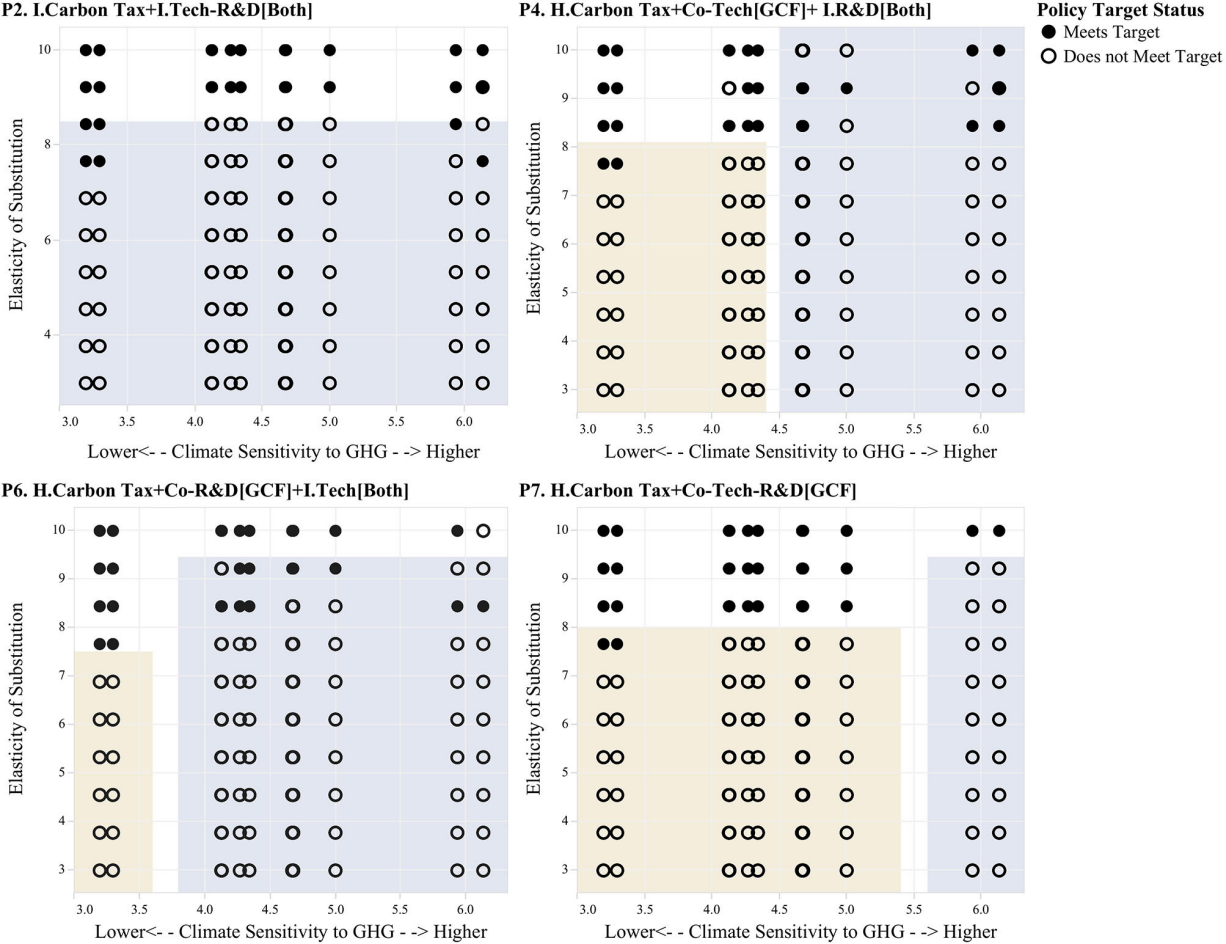
PRIM Boxes Describing Decision Relevant Scenarios. Filled circles show non-vulnerable cases, where the CO_2_ stabilization and temperature rise targets are met, and open circles indicate futures in which one of these two targets is not met. These futures are plotted across the two uncertainty dimensions that are found to be most relevant using PRIM: (1) the elasticity of substitution and (2) the climate sensitivity to GHG.

We first use scenario discovery to understand the cases in which end-of-century CO_2_ stabilization at 2°C targets is not met. These are futures in which CO_2_ stabilization is not achieved and in which end-of-century temperature rise is above 2°C. Figure 8 shows the results of this clustering analysis. The figure shows a series of scatter plots of all futures for different policy regimes. Filled circles show non-vulnerable cases, where the CO_2_ stabilization and temperature rise targets are met, and open circles indicate futures in which one of these two targets is not met. These futures are plotted across the two uncertainty dimensions that are found to be most relevant using PRIM: (1) the elasticity of substitution and (2) the climate sensitivity to GHG. High values of the elasticity of substitution describe scenarios in which the technologies across sectors are highly substitutable, which are more favorable for climate policy. Low values of the elasticity of substitution denote scenarios in which sectors are less substitutable, which makes it harder to move away from fossil energy. For the case of climate sensitivity, high values describe climate scenarios in which global temperature rises rapidly with growing CO_2_, thus making it harder to keep temperature levels below the 2°C target. Low values are associated with climate scenarios for which global temperature rises less abruptly with growing CO_2_ emissions. Finally, the shaded regions highlighted in yellow and blue were selected using scenario discovery to describe these sets of vulnerable futures. Table 5 provides a detailed description of the boundary conditions of each scenario box, as well as the corresponding coverage and density statistics that describe to which extend these scenario boxes adequately capture the vulnerable conditions of each policy.

**Table 5:** Scenario discovery analysis summary results for stabilization target.

**Policy name**	**Scenario box**	**Scenario description**	**Coverage**	**Density**
P2. I. Carbon Tax + I. Tech-R&D[Both]	Box1	• Elasticity of substitution <9.0	99% (445/447)	93% (413/447)
P4. H. Carbon Tax + Co-Tech[GCF] + I. R&D[Both]	Box1	• Climate sensitivity to GHG > 4.5	53% (237/447)	80% (190/237)
	Box2	• Elasticity of substitution <8.0 • Climate sensitivity to GHG <4.5	45% (202/447)	95% (182/202)
P6. H. Carbon Tax + Co-R&D[GCF] + I.Tech[Both]	Box1	• Elasticity of substitution <9.5 • Climate Sensitivity to GHG > 4.0	86% (392/455)	87% (341/392)
	Box2	• Elasticity of substitution <7.6 • Climate sensitivity to GHG <4.0	13% (60/455)	100% (60/60)
P7. H. Carbon Tax + Co-Tech-R&D[GCF]	Box1	• Elasticity of substitution <9.5 • Climate sensitivity to GHG > 5.5	30% (130/435)	97% (126/435)
	Box2	• Elasticity of substitution <8.0 • Climate sensitivity to GHG > 5.5	70% (305/435)	97% (296/305)

*The table summarizes the statistical properties (i.e., coverage and density) of the scenario boxes describing the vulnerability conditions of each policy regime. The quantitative thresholds defining each scenario box are listed*.

The results presented in Figure 8 and Table 5 show that the vulnerability region varies slightly across the different environmental policy regimes. For the independent comprehensive policy (“*P2 I. Carbon Tax*+*I. Tech-R&D[Both]*”), the vulnerability region is defined solely by the elasticity of substitution. The optimal policy under this regime fails to meet the stabilization target in all scenarios that do not display a high elasticity of substitution. For the other three policy regimes, the vulnerability region is described by both the elasticity of substitution and climate sensitivity. Scenario box 1 describes “high climate sensitivity futures,” while Scenario box 2 describes “medium-to-low elasticity of substitution scenarios.” Differences in the vulnerable region exists between these three policy architectures, namely, that the comprehensive GCF policy (“*P7. H. Carbon Tax* + *Co-Tech-R&D[GCF]*”) shows a greater area of success than the other three policy architectures.

These results also show that out of the four uncertainties considered in this analysis, (1) elasticity of substitution, (2) climate sensitivity, (3) atmospheric carbon sink capacity, and (4) the discount rate, only the first two determine whether or not the optimal policy achieves the objective of stabilizing CO_2_ emissions at sustainable levels before the end of the century. Arguably, out of these two factors, the elasticity of substitution plays a more fundamental role in determining the vulnerability of the policy response, as all scenarios that display medium to low elasticity of substitution are vulnerable across all policy regimes, while high climate sensitivity scenarios induce vulnerability at high elasticity of substitution scenarios for three out of the four policy regimes considered.

On the other hand, the end-of-century 2°C temperature rise target is met in a greater number of futures than the stabilization target. This implies that the former is a more achievable target than the later. Certainly, meeting the stabilization target would be highly beneficial as this would imply that climate change would not be a prevailing public policy problem after the end of the century; however, the results show that this target is met only under very favorable economic and environmental circumstances.

## Discussion

### Key Lessons From the Case Study

The results presented in the previous sections show that the combined application of multiple computational intelligence tools produces new insights and more detailed information about the effectiveness of different climate policy regimes. First, the use of the EDIAM model allows for the joint consideration of multiple regions and the interaction between the economy, the environment, and optimal climate policy. As a result, it is possible to analyze climate change policy multidimensionally in terms of both its ability to mitigate temperature rise and its economic cost (or benefit). Second, by using the EDIAM model in a computational experimentation setting, we show that an uncoordinated carbon tax is the highest cost policy in the majority of cases and that interregional cooperation through the GCF can sometimes be more costly than independent comprehensive climate policy. Our experiment also highlights that there are noticeable differences between policies in terms of the period of time required to achieve stabilization (cooperation between regions generally induces decarbonization faster than non-cooperation). However, we also find that for a considerable number of futures, policy intervention needs to remain in place for as long as 300 years.

Through the application of data visualization techniques, we show that it is possible to describe the dynamics of optimal climate regulation. It doing so, we find that regional differences play a significant role in determining the structure of the optimal policy response. Particularly, we show that in emerging economies carbon taxation is always equal or higher than carbon taxation in advanced economies. In contrast, the technology policy effort of climate policy is stronger in advanced economies than in the emerging economies. Since mitigation technologies are mainly produced in advanced nations, then the optimal policy prioritizes accelerating technology development over taxation in this region, while in the emerging region, higher taxation creates a strong market niche for sustainable energy diffusion, which is used more effectively by R&D and technology subsidies that accelerate the technological catching-up process.

We demonstrate that it is possible to use clustering algorithms to quantitatively identify key drivers of vulnerability of climate policy across various objectives. We find that out of the four stressors considered, (1) elasticity of substitution, (2) climate sensitivity to GHG emissions, (3) discount rate of economic agents, and (4) carbon sink capacity, only the first two determine whether or not the optimal policy achieves the objective of stabilizing CO_2_ emissions at sustainable levels before the end of the century. Considering the relevance of the debate about the appropriate value of the discount rate in climate policy analysis, this finding, which shows that there are more critical drivers of climate policy vulnerability, exemplifies very well the benefits of combining different computational tools for decision analysis in complex systems. Finally, we show that for the independent carbon taxes policy (i.e., P1), in the majority of cases, it is possible to keep the temperature rise below 2°C, but in none of the cases, this policy is able to stabilize CO_2_ emissions before the end of the century.

### A Hierarchy of Computational Tools for Analyzing Sustainability Challenges

**Figure 9:**
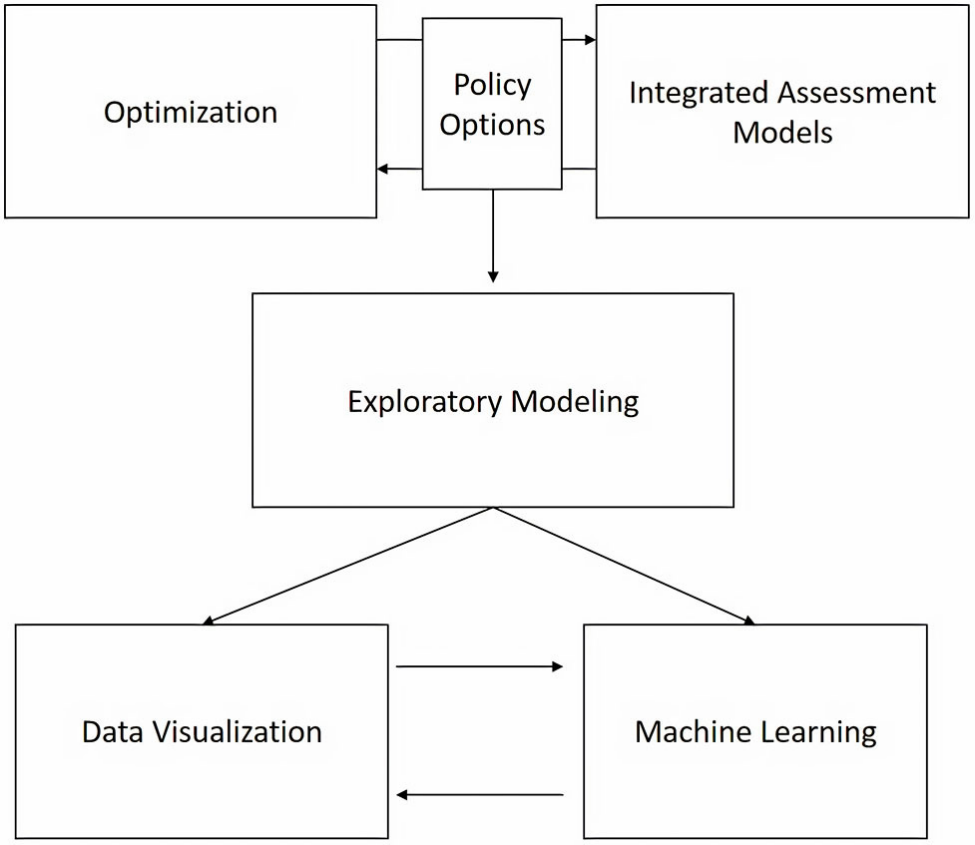
Hierarchical Relationship Between Computational Tools.

The combined application of various computational tools to this case study yields lessons with respect to their hierarchical relation for analyzing sustainability challenges amid complexity and deep uncertainty. Figure 9 describes this hierarchy schematically; each block represents an analytical element to be integrated in the analysis of sustainability challenges, and arrows indicate information flows in this hierarchy.

As shown in this case study, the first layer in this hierarchy englobes optimization and Integrated Assessment Models (IAMs). The combination of both perspectives is conducive for analyzing sustainability challenges. IAMs provide the required formalism and tractability for taking into consideration sustainability interdependencies across spheres. Optimization provides the analytical framework needed for formalizing policy options in the light of sustainability objectives. This requires adequate cost estimates of competing alternatives, formalization of decision restrictions, and sustainability performance metrics for all systems considered. The second layer pertains to the integration of the models produced in the first layer with exploratory modeling (Bankes, 1993; Kwakkel, 2017). The intention of using exploratory modeling is to produce, for each parametrization case, a vector of optimal action. This yields a rich database that maps out changes in optimal action across the often vast ensemble of cases considered. The third layer of this hierarchy connects with the second by the direct application of data visualization and machine learning techniques. Machine learning techniques, in particular clustering techniques and decision rule classifiers, can be used to identify statistically (a) vulnerability conditions of sustainability objectives across policy alternatives and (b) critical thresholds for triggering different actions. Data visualization techniques can be particularly useful to track down changes of the optimal policy response across the parameterization space and to create decision-support tools to be used in participatory planning exercises.

This integration of computational tools is useful because the statistical evidence produced through the integration of these tools leads to a more nuanced understanding of the conditions under which different policy alternatives are more appropriate for achieving sustainability goals. For example, Molina-Perez et al. (2019) apply a similar approach for analyzing sustainability water challenges amid climate, economic, and technological deep uncertainty. In their analysis, the authors integrate econometric, water, and climate modeling tools to develop an IAM, which is combined with an optimization framework that assesses how to best expand the water infrastructure of Monterrey, Mexico. Their results show that it is possible to develop a robust expansion strategy that meets systems' reliability and environmental restrictions without exposing the city to large financial and operational risks. Such strategy is comprised of a diversified collection of projects that considers both conventional and non-conventional expansion strategies and that postpones large infrastructure investment until more information about climate and technological change becomes available.

There are multiple avenues for future research with respect to integrating multiple computational tools for analyzing sustainability challenges. On the one hand, this line research will greatly benefit from standard statistical procedures for designing experimental designs that reduce the risks of biases and increase precision of estimations. This is challenging as each one of these tools (i.e., simulation models, optimization, and machine learning algorithms) needs to be calibrated, trained, and parametrized. In current studies, this is mainly done *ad hoc* and there is little evidence describing, for example, how parameter selection in an optimization routine impacts statistical inference of a classification algorithm; the same is true for experimental designs in exploratory modeling exercises. On the other hand, there is ample room for studying, from a behavioral perspective, how to best transfer findings of these studies to non-specialized audiences. For instance, experimental evidence comparing the impact on knowledge transfer of different combinations of computational tools could shed light on the most appropriate approach for integration.

## Conclusions

This paper applies DMDU methods to structure an analysis of global climate change mitigation and to demonstrate that the combination of multiple computational tools for analyzing this sort of sustainability challenges leads to richer analytical insights than those produced by traditional monodisciplinary studies.

The scope of the computational experiment in the study considers nine different policy regimes and 600 different optimization cases. The ensemble of cases combines four sources of uncertainty: elasticity of substitution, discount rate, climate sensitivity to GHG, and atmospheric carbon sink capacity. The performance of the different policy regimes is evaluated in terms of the end-of-century conditions. Particularly, the performance of each policy regime is evaluated in terms of its capacity to meet two climate change sustainability objectives: (1) the stabilization of CO2 emissions and (2) the 2°C temperature rise target.

The analysis shows that the structure of optimal environmental regulation changes markedly across the uncertainty space. The results show that the optimal policy response is most affected by climate sensitivity uncertainty and the elasticity of substitution uncertainty. In particular, the strength of the optimal policy response is directly proportional to the level of climate sensitivity to greenhouse gas emissions and inversely proportional to the elasticity of substitution between the sustainable energy and fossil energy sectors. We also show that the discount rate does affect the structure of the optimal policy response, but its influence is less significant when compared to the influence of climate sensitivity and the elasticity of substitution.

The comparison of GCF-based policy regimes and non-GCF policy regimes shows that the GCF does affect the structure of climate policy. These results show that under the GCF the level of carbon taxation reduces for both regions compared to the level of taxation in the non-cooperative policy regimes. Also under the GCF, the optimal level of effort in R&D and technology subsidies in the emerging region is on average higher than the optimal level of effort in the non-cooperative policy regime. This indicates that under the GCF it is feasible for the emerging region to make higher investments in R&D and technology subsidies and reduce the rate of taxation. Similarly, for the advanced region it is shown that it is possible to reduce the level of carbon taxation by co-funding R&D and technology subsidies in the emerging region.

We use machine learning algorithms to analyze the experimental database. These results show that the objective stabilizing CO_2_ emissions below 2°C before the end of the century is rarely met. Two decision relevant clusters describe this type of vulnerability: (1) high climate sensitivity to greenhouse gas emissions and (2) medium-low elasticity of substitution. In contrast, the 2°C temperature rise target without CO_2_ stabilization is met in a greater number of cases. For both types of vulnerability, the role of discount rate in defining the vulnerability conditions is found to be minimal.

This analysis shows that by integrating optimization, complex simulation models, and machine learning algorithms, it is possible to quantitatively identify key drivers of vulnerability of climate change mitigation policies. Drawing on lessons from this case study, we propose an analytical hierarchy of computational tools that can be applied to other sustainability challenges. The first layer of this hierarchy consists of coupling IAMs with optimization to capture sustainability interdependencies across systems and path dependencies of optimal policy decisions. The second layer proposes to use exploratory modeling (Bankes, 1993; Kwakkel, 2017) to deal with deep uncertainty. Finally, the third layer of this hierarchy connects with the second by the direct application of data visualization and machine learning techniques for identifying relevant decision clusters, characterizing vulnerability conditions, and identifying critical sustainability thresholds.

## Data Availability Statement

All datasets generated for this study are included in the article/Supplementary Material.

## Author Contributions

EM-P developed the mathematical model used in this study and lead the analysis of experimental results. OE-F developed the computational architecture needed to run the experiment in a cloud computer cluster. HZ-M developed the connection of this study to sustainability sciences and collaborated in the analysis of experimental results. All authors contributed to the article and approved the submitted version.

## Conflict of Interest

The authors declare that the research was conducted in the absence of any commercial or financial relationships that could be construed as a potential conflict of interest.

## Footnotes

^1^The complete specification of EDIAM, including the derivation of intertemporal dynamics and calibration, can be found in Molina-Perez (2016), specifically Chapter 3, pages 31–59, Appendix A, pages 152–161, and Appendix C, pages 163 and 164. Additionally, we have made publicly available all datasets and programming scripts describing the operationalization of EDIAM's framework in the following github repository: https://github.com/emolinaperez/Ediam_vFrontiers.

^2^The EDIAM model is specified in continuous form. Yet, it is important to note that the discounted values of utility are computed using discrete time steps after the continuous model is numerically solved.

^3^For clarity, all time subscripts are omitted, but all variables in this optimization set up are dynamic.

^4^The model is not defined beyond this limit of temperature rise because such level of temperature rise will result in abrupt and irreversible changes to the global climate system, including events such as the ice sheet collapse, permafrost carbon release, and methane hydrate release (IPCC, 2013).
