# Supplementary material for: Computational Intelligence for Studying Sustainability Challenges: Tools and Methods for Dealing With Deep Uncertainty and Complexity
Source: Front Robot AI. 2020 Sep 17;7:111. doi: 10.3389/frobt.2020.00111 (PMC7805846; doi:10.3389/frobt.2020.00111)
Supplement: Supplementary file 2 [file Data_Sheet_2.ZIP › ClimateDataCalibration/CMIP5_modeling_groups.pdf]

Modeling Groups and their Terms of Use

The "official" model and group names given in the table below should be used in all presentations and publications (e.g., in tables and figure legends).

Output from yellow highlighted models is available for unrestricted use. Output from the others may only be used for non-commercial research and educational purposes. [See complete "Terms of Use": http://cmip-pcmdi.llnl.gov/cmip5/terms.html]

Modeling Center (or Group)	Institute ID	Model Name
Commonwealth Scientific and Industrial Research Organization (CSIRO) and Bureau of Meteorology (BOM), Australia	CSIRO-BOM	ACCESS1.0<br>ACCESS1.3
Beijing Climate Center, China Meteorological Administration	BCC	BCC-CSM1.1<br>BCC-CSM1.1(m)
Instituto Nacional de Pesquisas Espaciais (National Institute for Space Research)	INPE	BESM OA 2.3*
College of Global Change and Earth System Science, Beijing Normal University	GCESS	BNU-ESM
Canadian Centre for Climate Modelling and Analysis	CCCMA	CanESM2<br>CanCM4<br>CanAM4
University of Miami - RSMAS	RSMAS	CCSM4(RSMAS)*
National Center for Atmospheric Research	NCAR	CCSM4
Community Earth System Model Contributors	NSF-DOE-NCAR	CESM1(BGC)<br>CESM1(CAM5)<br>CESM1(CAM5.1,FV2)<br>CESM1(FASTCHEM)<br>CESM1(WACCM)
Center for Ocean-Land-Atmosphere Studies and National Centers for Environmental Prediction	COLA and NCEP	CFSv2-2011
Centro Euro-Mediterraneo per I Cambiamenti Climatici	CMCC	CMCC-CESM<br>CMCC-CM<br>CMCC-CMS
Centre National de Recherches Météorologiques / Centre Européen de Recherche et Formation Avancée en Calcul Scientifique	CNRM-CERFACS	CNRM-CM5<br>CNRM-CM5-2
Commonwealth Scientific and Industrial Research Organization in collaboration with Queensland Climate Change Centre of Excellence	CSIRO-QCCCE	CSIRO-Mk3.6.0
EC-EARTH consortium	EC-EARTH	EC-EARTH
LASG, Institute of Atmospheric Physics, Chinese Academy of Sciences and CESS,Tsinghua University	LASG-CESS	FGOALS-g2

* Model output not yet available.

LASG, Institute of Atmospheric Physics, Chinese Academy of Sciences	LASG-IAP	FGOALS-gl<br>FGOALS-s2
The First Institute of Oceanography, SOA, China	FIO	FIO-ESM
NASA Global Modeling and Assimilation Office	NASA GMAO	GEOS-5
NOAA Geophysical Fluid Dynamics Laboratory	NOAA GFDL	GFDL-CM2.1<br>GFDL-CM3<br>GFDL-ESM2G<br>GFDL-ESM2M<br>GFDL-HIRAM-C180<br>GFDL-HIRAM-C360
NASA Goddard Institute for Space Studies	NASA GISS	GISS-E2-H<br>GISS-E2-H-CC<br>GISS-E2-R<br>GISS-E2-R-CC
National Institute of Meteorological Research/Korea Meteorological Administration	NIMR/KMA	HadGEM2-AO
Met Office Hadley Centre (additional HadGEM2-ES realizations contributed by Instituto Nacional de Pesquisas Espaciais)	MOHC (additional realizations by INPE)	HadCM3<br>HadGEM2-CC<br>HadGEM2-ES<br>HadGEM2-A
Institute for Numerical Mathematics	INM	INM-CM4
Institut Pierre-Simon Laplace	IPSL	IPSL-CM5A-LR<br>IPSL-CM5A-MR<br>IPSL-CM5B-LR
Japan Agency for Marine-Earth Science and Technology, Atmosphere and Ocean Research Institute (The University of Tokyo), and National Institute for Environmental Studies	MIROC	MIROC-ESM<br>MIROC-ESM-CHEM
Atmosphere and Ocean Research Institute (The University of Tokyo), National Institute for Environmental Studies, and Japan Agency for Marine-Earth Science and Technology	MIROC	MIROC4h<br>MIROC5
Max-Planck-Institut für Meteorologie (Max Planck Institute for Meteorology)	MPI-M	MPI-ESM-MR<br>MPI-ESM-LR<br>MPI-ESM-P
Meteorological Research Institute	MRI	MRI-AGCM3.2H<br>MRI-AGCM3.2S<br>MRI-CGCM3<br>MRI-ESM1
Nonhydrostatic Icosahedral Atmospheric Model Group	NICAM	NICAM.09
Norwegian Climate Centre	NCC	NorESM1-M<br>NorESM1-ME
